# Complete Genome Sequence of *Citrobacter freundii* 705SK3, an OXA-48-Encoding Wastewater Isolate

**DOI:** 10.1128/genomeA.00842-17

**Published:** 2017-08-17

**Authors:** Katrin Zurfluh, Roger Stephan, Jochen Klumpp, Magdalena Nüesch-Inderbinen, Jörg Hummerjohann, Claudia Bagutti, Roger Marti

**Affiliations:** a Institute for Food Safety and Hygiene, Vetsuisse Faculty University of Zurich, Zurich, Switzerland; b Institute of Food, Nutrition and Health, ETH Zurich, Zurich, Switzerland; c Division of Food Microbial Systems, Microbiological Safety of Foods of Animal Origin Group, Agroscope, Bern, Switzerland; d Biosafety Laboratory, State Laboratory Basel-City, Basel, Switzerland

## Abstract

We present the genome sequence of *Citrobacter freundii* 705SK3, a wastewater isolate harboring an IncL OXA-48-encoding plasmid. Assembly of the genome resulted in a 5,242,839-bp circular chromosome (GC content, 52%) and two closed plasmids of 296,175 bp and 63, 458 bp in size.

## GENOME ANNOUNCEMENT

*Citrobacter freundii* is an opportunistic pathogen and is frequently found in the environment (water and soil), but it has also been isolated from food and the intestines of animals and humans. Although its virulence potential is rather low, *C. freundii* can be the causative agent of a wide spectrum of infections involving the gastrointestinal, urinary, or respiratory tract or even the central nervous system (1). *C. freundii* possesses an inducible AmpC β-lactamase, which can pose a challenge for antibiotic susceptibility reporting because an *in vitro* susceptibility may not correlate with clinical efficacy (2). With the general increase in the number of carbapenemase-producing *Enterobacteriaceae*, reports of carbapenemase-producing *C. freundii* have also increased (3–6). The main carbapenemases produced by *Enterobacteriaceae* belong to the Ambler class A (e.g., KPC), class B (e.g., IMP, VIM, and NDM) or class D (e.g., OXA-48 and its variants). Class D β-lactamases generally possess weak but significant carbapenemase activity (7). However, the combination of an (inducible) AmpC β-lactamase together with an OXA-48 makes such isolates resistant against almost all β-lactams available.

Here, we present the genome sequence of an OXA-48-producing *C. freundii* isolate collected from wastewater near Basel, Switzerland, in December 2015 (8). The genome was sequenced at the Functional Genomics Center Zurich (FGCZ) using Pacific Biosciences (PacBio) single-molecule real-time (SMRT) technology RS2 reads (C4/P6 chemistry). *De novo* assembly was performed using SMRTAnalysis 2.3 with the HGAP3 protocol. Annotation was done using the NCBI Prokaryotic Genome Annotation Pipeline (9). The MLST-1.8 server (10), ResFinder 2.1 (11), and PlasmidFinder 1.3 (12) were used to identify sequence type (ST), acquired resistance genes, and plasmid incompatibility types.

The assembly resulted in one chromosome and two plasmids (all sequences were closed). The chromosome is 5,242,839 bp in size (GC content, 52%) and encodes the AmpC β-lactamase CMY-75. The isolate could not be assigned to an existing ST, although it is highly similar to ST112 (only one point mutation in the *fadD* gene: allele 69 position 363G → A). The larger of the two plasmids, p705SK3_1, is 296,175 bp in size with a GC content of 47.8% and does not contain any antimicrobial resistance genes. PlasmidFinder was not able to assign this plasmid to any plasmid incompatibility group. The second plasmid (63,458 bp, 51.2% GC content), p705SK3_2, belongs to the incompatibility type IncL and carries the *bla*_OXA-48_ gene. It shows remarkable similarities to the prototype of the IncL plasmids involved in the worldwide spread of OXA-48 (7, 13). The main difference was the presence of two IS*1*R elements on p705SK3_2 compared to pOXA-48 (GenBank accession number JN626286). The first IS*1*R is located 163 bp downstream of the *bla*_OXA-48_ gene, and the second is 380 bp downstream of *korC*, which encodes a hypothetical transcriptional repressor. Furthermore, p705SK3_2 possesses 99.96% identity to p704SK10_2 (CP022150), an OXA-48-encoding IncL plasmid extracted from an *Enterobacter cloacae* isolate from the same wastewater (14).

The isolate *C. freundii* 705SK3 provides further proof that *Citrobacter* species are a potential reservoir for the widely disseminated OXA-48-encoding IncL plasmids.

### Accession number(s).

Sequence and annotation data of the genome have been deposited in GenBank under accession numbers CP022151 (chromosome), CP022152 (p705SK3_1), and CP022153 (p705SK3_2). This is the first version of this genome.
